# A Systematic Review of Catatonia Following Hypoxic-Ischaemic Brain Injury in Adults

**DOI:** 10.1192/bjo.2026.11249

**Published:** 2026-06-30

**Authors:** Sirous Golchinheydari

**Affiliations:** South London and Maudsley NHS Foundation Trust, London, United Kingdom

## Abstract

**Aims:**

Catatonia is a treatable syndrome that can occur due to psychiatric and neurological pathology, such as hypoxic-ischaemic brain injury. Many neuroanatomical pathways may contribute to catatonia but no single pathway has been found to be the sole cause.

A systematic review assessing evidence of adult-only cases of catatonia after hypoxic-ischaemic brain injury and summarisation of the presentation, lesion location, treatment and outcome.

**Methods:**

A PRISMA-2020 compliant PubMed/MEDLINE search of terms related to catatonia and hypoxia/ischaemia was carried out. Catatonia had to be explicitly diagnosed and must have been secondary to a hypoxic-ischaemic insult. Data were extracted based on lesion characteristics, timeline, treatment, and outcome.

**Results:**

Seven studies met the criteria and they were all case reports. Lesions were heterogeneous and included bilateral parietal watershed infarcts, basal ganglia, thalamus,corona radiata, extensive white matter disease, pontine and cerebellar infarcts, and fronto-insular atrophy with inferred hypoxia. Presentations ranged from stuporous catatonia to delirium and affective disorders with catatonic features. Outcomes were generally good and catatonia resolved; however, post-neurological consequences of hypoxic-ischaemic insult remained in a few.

**Conclusion:**

The evidence was limited to case reports and no single lesion causing catatonia was found.The findings supported the disrupted cortico-striato-thalamo-cortical motor pathway and the disconnection pathway as a possible cause for catatonia. There was also evidence for the involvement of neurochemical pathways such as GABA, dopamine and NMDA as a potential cause or treatment focus. Further studies with standardised catatonia measures and imaging are required.

